# *C9ORF72* GGGGCC Expanded Repeats Produce Splicing Dysregulation which Correlates with Disease Severity in Amyotrophic Lateral Sclerosis

**DOI:** 10.1371/journal.pone.0127376

**Published:** 2015-05-27

**Authors:** Johnathan Cooper-Knock, Joanna J. Bury, Paul R Heath, Matthew Wyles, Adrian Higginbottom, Catherine Gelsthorpe, J. Robin Highley, Guillaume Hautbergue, Magnus Rattray, Janine Kirby, Pamela J. Shaw

**Affiliations:** 1 Sheffield Institute for Translational Neuroscience (SITraN), University of Sheffield, 385A Glossop Road, Sheffield, S10 2HQ, United Kingdom; 2 Life Sciences, The University of Manchester, Michael Smith Building, Oxford Road, Manchester, M13 9PT, United Kingdom; National Institute of Health, UNITED STATES

## Abstract

**Objective:**

An intronic GGGGCC-repeat expansion of *C9ORF72* is the most common genetic variant of amyotrophic lateral sclerosis (ALS) and frontotemporal dementia. The mechanism of neurodegeneration is unknown, but a direct effect on RNA processing mediated by RNA foci transcribed from the repeat sequence has been proposed.

**Methods:**

Gene expression profiling utilised total RNA extracted from motor neurons and lymphoblastoid cell lines derived from human ALS patients, including those with an expansion of *C9ORF72*, and controls. In lymphoblastoid cell lines, expansion length and the frequency of sense and antisense RNA foci was also examined.

**Results:**

Gene level analysis revealed a number of differentially expressed networks and both cell types exhibited dysregulation of a network functionally enriched for genes encoding ‘RNA splicing’ proteins. There was a significant overlap of these genes with an independently generated list of GGGGCC-repeat protein binding partners. At the exon level, in lymphoblastoid cells derived from *C9ORF72*-ALS patients splicing consistency was lower than in lines derived from non-C9ORF72 ALS patients or controls; furthermore splicing consistency was lower in samples derived from patients with faster disease progression. Frequency of sense RNA foci showed a trend towards being higher in lymphoblastoid cells derived from patients with shorter survival, but there was no detectable correlation between disease severity and DNA expansion length.

**Significance:**

Up-regulation of genes encoding predicted binding partners of the *C9ORF72* expansion is consistent with an attempted compensation for sequestration of these proteins. A number of studies have analysed changes in the transcriptome caused by *C9ORF72* expansion, but to date findings have been inconsistent. As a potential explanation we suggest that dynamic sequestration of RNA processing proteins by RNA foci might lead to a loss of splicing consistency; indeed in our samples measurement of splicing consistency correlates with disease severity.

## Introduction

GGGGCC repeat expansions within intron 1 of the *C9ORF72* gene are the most common cause of familial amyotrophic lateral sclerosis (ALS) and familial frontotemporal degeneration (FTD) [1,2], though how this genetic change results in neuronal injury is not yet understood. Evidence is being gathered for a gain-of-function toxicity mediated by either sequestration of RNA binding proteins (RBPs) by RNA foci transcribed from the repeat sequence [3–8], or via repeat associated non-ATG (RAN) translation of the repeat sequence to produce a dipeptide repeat protein [9–11], or a combination of both mechanisms.

Gene expression profiling has the potential to identify biological pathways aberrantly affected by the *C9ORF72* expansion. In addition, if toxicity is mediated by nuclear RNA foci developed from an intronic expansion, then transcriptome changes may be relatively upstream in disease pathogenesis [12]. On this basis we have studied gene expression changes in motor neurons and lymphoblastoid cell lines derived from individuals with *C9ORF72*-ALS.

We have previously suggested that dynamic sequestration by RNA foci of a number of RBPs might affect nuclear speckle function and thus disrupt mRNA splicing [8]. It has been proposed that splicing errors are a normal occurrence for which the cell is able to compensate [13]. Therefore an excessive splicing error rate may not immediately result in disease; however in time compensatory mechanisms might be overwhelmed in vulnerable cells. This is more consistent with the variable phenotype and late age of onset seen in *C9ORF72*-ALS than a model of binary toxicity resulting from a small number of specific splicing errors. Therefore we aimed to derive a measure of the overall splicing error rate in biosamples containing the *C9ORF72* repeat expansion. Additionally we used Southern hybridisation and RNA fluorescence in-situ hybridisation (FISH) to examine the relationship between the changes in the splicing error rate, disease severity, the length of the GGGGCC repeat expansion and the abundance of RNA foci.

## Results

### Transcriptome analysis

#### Motor neurons

**Fig 1:**
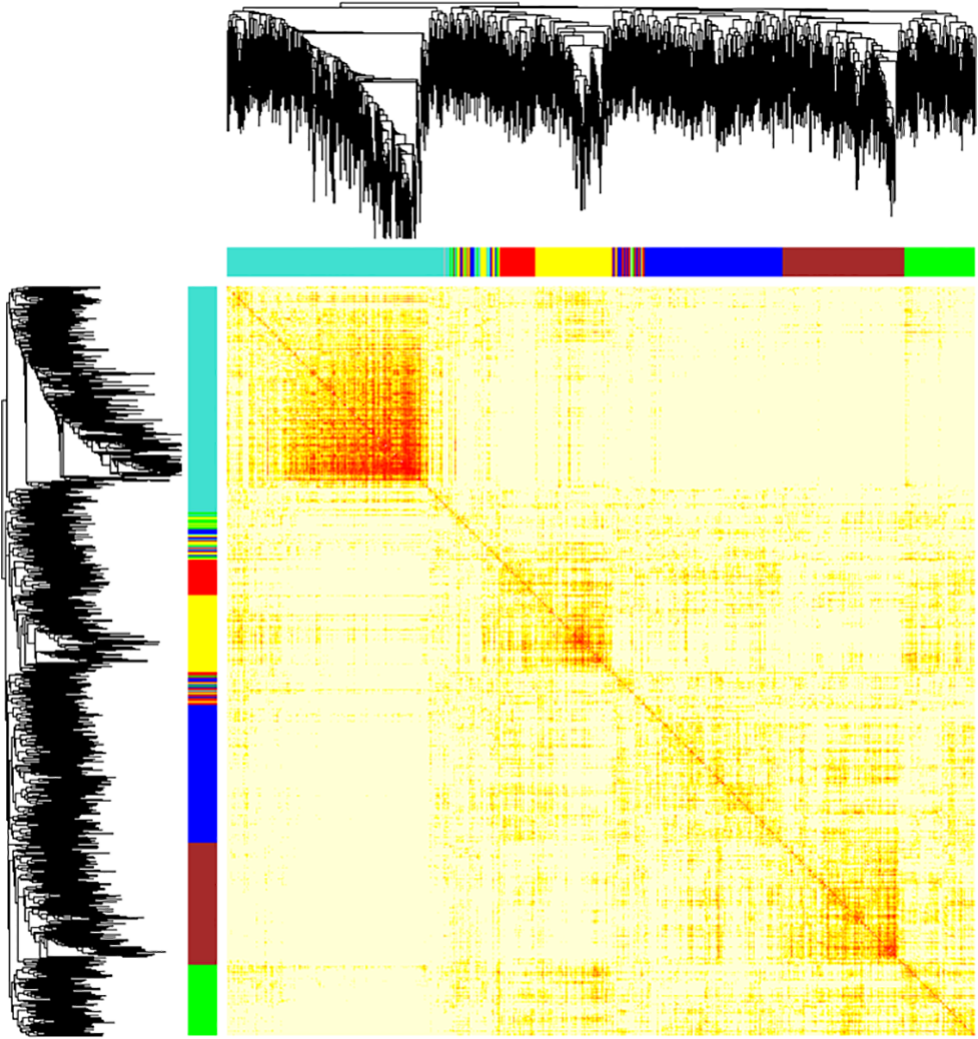
Gene level network analysis of transcriptome changes in motor neurons from *C9ORF72*-ALS cases. WGCNA analysis identified six gene networks which were dysregulated between *C9ORF72*-ALS and control samples. A clustering tree and heat map are shown illustrating separation of the gene networks, a lower branch height or darker colour denotes a greater Pearson correlation coefficient between pairs of genes.

Network analysis using WGCNA identified six significant networks within 5,000 genes considered (**Fig 1**) all of which were differentially expressed between *C9ORF72*-ALS and control groups, and showed significant functional enrichment (**Table 1**). Based on the median fold change, three networks were down-regulated and three networks were up-regulated in *C9ORF72*-ALS (**Table 1**). Specifically, within the brown network which was significantly enriched for transcripts related to the Gene Ontology (GO) term ‘RNA splicing’, 58.2% of transcripts were up-regulated. The yellow and green networks were also up-regulated and functionally enriched for ‘male sex differentiation’ and ‘erythrocyte homeostasis’ respectively. The turquoise, blue and red networks were all down-regulated and functionally enriched for ‘cholesterol biosynthetic process’, ‘regulation of glucose metabolic process’ and ‘regulation of nuclear division’ respectively.

**Table 1:** Gene level network analysis of transcriptome changes in motor neurons from *C9ORF72*-ALS cases.

Network	Number of Genes	P-value C9ORF72-ALS Vs Control	Top Gene Ontology Enrichment	P-value for Enrichment Analysis	Median Fold Change
Turquoise	1555	0.008	Cholesterol biosynthetic process	0.001	0.62
Blue	1020	0.003	Regulation of glucose metabolic process	0.01	0.47
Brown	901	0.008	RNA splicing	7.45E-04	1.49
Yellow	635	0.003	Male sex differentiation	0.02	1.91
Green	579	0.0005	Erythrocyte homeostasis	0.01	1.75
Red	321	0.006	Regulation of nuclear division	0.01	0.49

WGCNA analysis identified six gene networks which were dysregulated between *C9ORF72*-ALS and control samples. The median fold change of genes within each network and the functional enrichment of each of the gene networks is tabulated. A fold change of >1 equates to up-regulation and a fold change of <1 equates to down-regulation.

#### Lymphoblastoid cell lines

**Fig 2:**
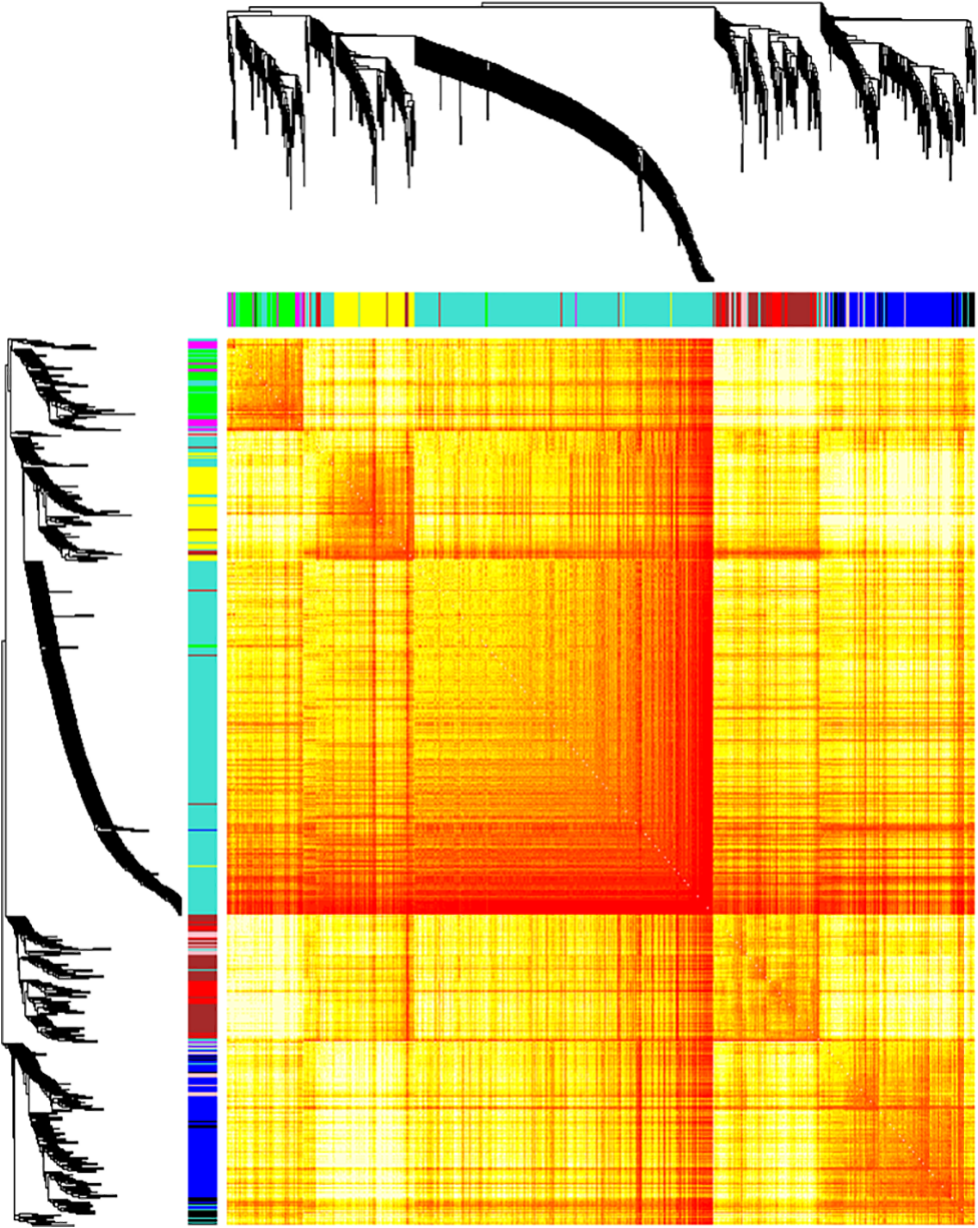
Gene level network analysis of transcriptome changes in lymphoblastoid cell lines derived from *C9ORF72*-ALS cases. WGCNA analysis identified nine gene networks which were dysregulated between *C9ORF72*-ALS and control samples. A clustering tree and heat map are shown illustrating separation of the gene networks, a lower branch height or darker colour denotes a greater Pearson correlation coefficient between pairs of genes. The median fold change of genes within each network and the functional enrichment of each of the gene networks is tabulated (B). A fold change of >1 equates to up-regulation and a fold change of <1 equates to down-regulation.

Two samples failed the Affymetrix quality control (QC) assessment and were excluded from the analysis based upon a low % presence call and/or AUC value. Network analysis using WGCNA identified nine significant networks which were differentially expressed between *C9ORF72*-ALS and control groups (**Fig 2**), and showed significant functional enrichment (**Table 2**). Based on the median fold change, five networks were down-regulated and four networks were up-regulated in *C9ORF72*-ALS (**Table 2**). Specifically 92% of transcripts were up-regulated within the green network, which was significantly enriched for transcripts related to the GO term ‘RNA splicing’. The turquoise and red networks were both functionally enriched for genes related to ‘positive regulation of apoptosis’, the blue and yellow networks were functionally enriched for categories directly related to neuronal function, and the black network was functionally enriched for ‘striated muscle tissue development’; the pink network was functionally enriched for ‘inflammatory response’; and the brown and magenta networks were functionally enriched for ‘protein catabolic process.’

**Table 2:** Gene level network analysis of transcriptome changes in lymphoblastoid cell lines derived from C9ORF72-ALS cases.

Network	Number of Genes	P-value C9ORF72-ALS Vs Control	Top Gene Ontology Enrichment	P-value for Enrichment Analysis	Median Fold Change
Turquoise	4653	8.11E-64	Positive regulation of apoptosis	0.01	1.19
Blue	1403	0.0000001	Regulation of action potential in neuron	0.02	0.88
Brown	1038	4.69E-09	Protein catabolic process	0.002	0.79
Yellow	854	2.48E-09	Synaptic transmission	0.004	1.16
Green	537	0.0000002	RNA splicing	1.50E-05	1.27
Red	427	9.17E-08	Positive regulation of apoptosis	0.02	0.74
Black	391	3.43E-09	Striated muscle tissue development	0.02	0.86
Pink	367	3.54E-08	Inflammatory response	0.004	0.86
Magenta	336	0.0000001	Protein catabolic process	1.43E-05	1.53

WGCNA analysis identified nine gene networks which were dysregulated between C9ORF72-ALS and control samples. The median fold change of genes within each network and the functional enrichment of each of the gene networks is tabulated. A fold change of >1 equates to up-regulation and a fold change of <1 equates to down-regulation.

#### Analysis of networks enriched for the GO term ‘RNA Splicing’

The brown network in the motor neurons and the green network in the lymphoblastoid cell lines were both up-regulated in *C9ORF72*-ALS samples and significantly enriched for transcripts related to the GO term ‘RNA splicing.’ We set out to determine whether the two networks contained similar transcripts or only transcripts with similar functional enrichment.

To make this comparison we reverted to the original lists of transcripts not filtered by Pearson correlation coefficient because computational burden was no longer an issue, and we were interested in all transcripts associated with the network signal and not just the most correlated. Examination of all genes significantly correlated (as quantified by Pearson correlation coefficient) (p<0.05) with the brown network signal and associated with the GO term ‘RNA Processing’ in motor neurons derived from *C9ORF72*-ALS patients revealed 88 transcripts encoding 74 unique genes (**S1 Table**). Examination of all genes significantly correlated (p<0.05) with the green network signal and associated with the GO term ‘RNA Processing’ in lymphoblastoid cells derived from *C9ORF72*-ALS patients revealed 459 transcripts encoding 236 unique genes (**S1 Table**). Given the difference in cell types and microarray platforms, there was evidence for significant similarity between the lists: 54% of the motor neuron list was also present within the lymphoblastoid cell list. Previously we have identified candidate binding partners of the GGGGCC repeat expansion by RNA pulldown and mass spectroscopy [8]. 20% of the unique hits identified in this way were present within the lymphoblastoid cell line list (p<0.0001) of which 89% were up-regulated in the *C9ORF72*-ALS samples compared to controls (**S1 Table**); and 10% of the unique hits were present within the motor neuron list (p<0.0001) of which 77% were up-regulated in the *C9ORF72*-ALS samples compared to controls (**S1 Table**).

#### Analysis of splicing

**Fig 3:**
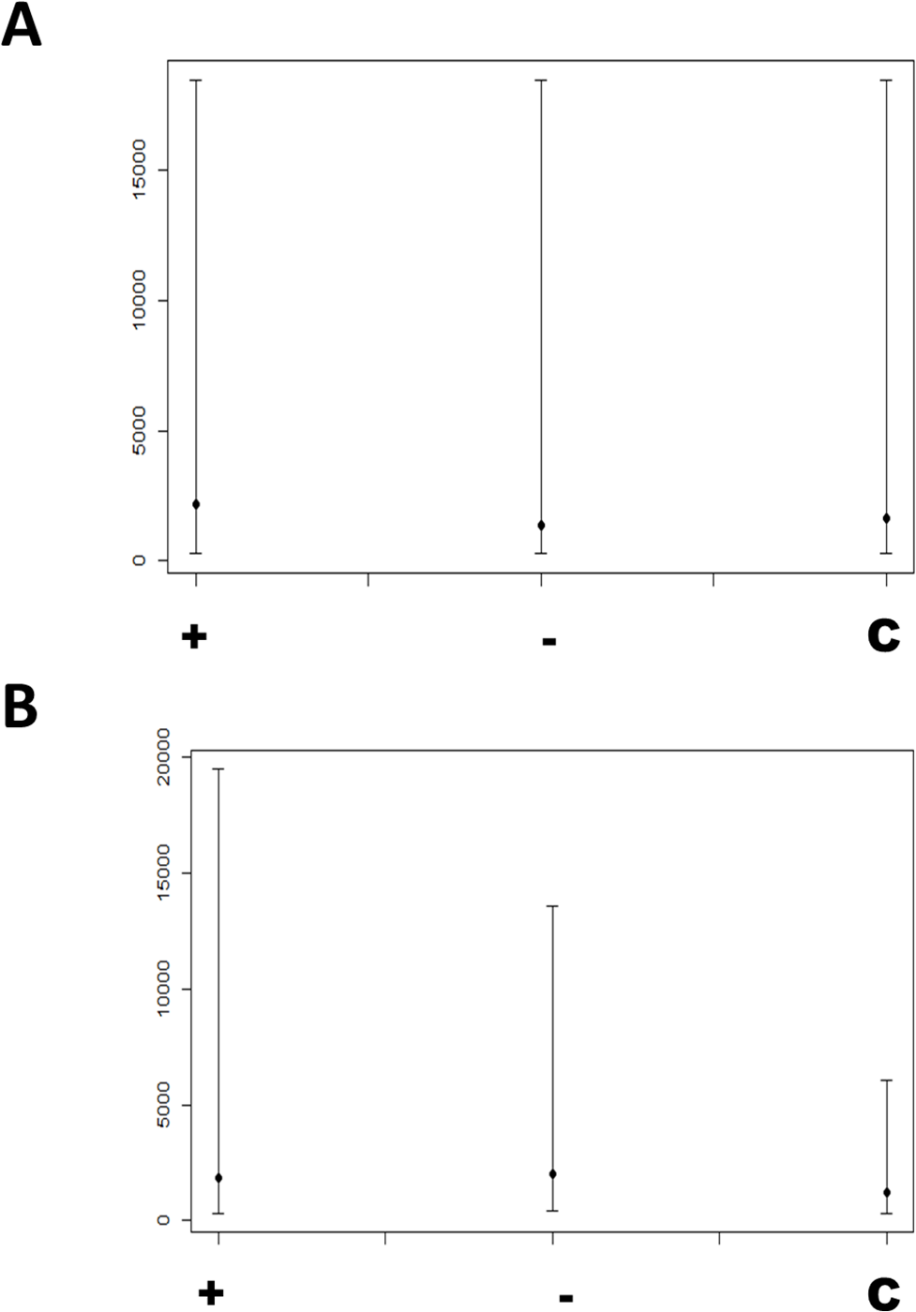
Frequency of exon inclusion and exclusion events. Plots of median and 95% CI for numbers of (A) exon inclusion and (B) exon exclusion events in *C9ORF72*-ALS (+), non-*C9ORF72* ALS (-) and control (C) derived lymphoblastoid cell lines, as determined by FIRMA score. There was no significant difference between sample groups.

**Fig 4:**
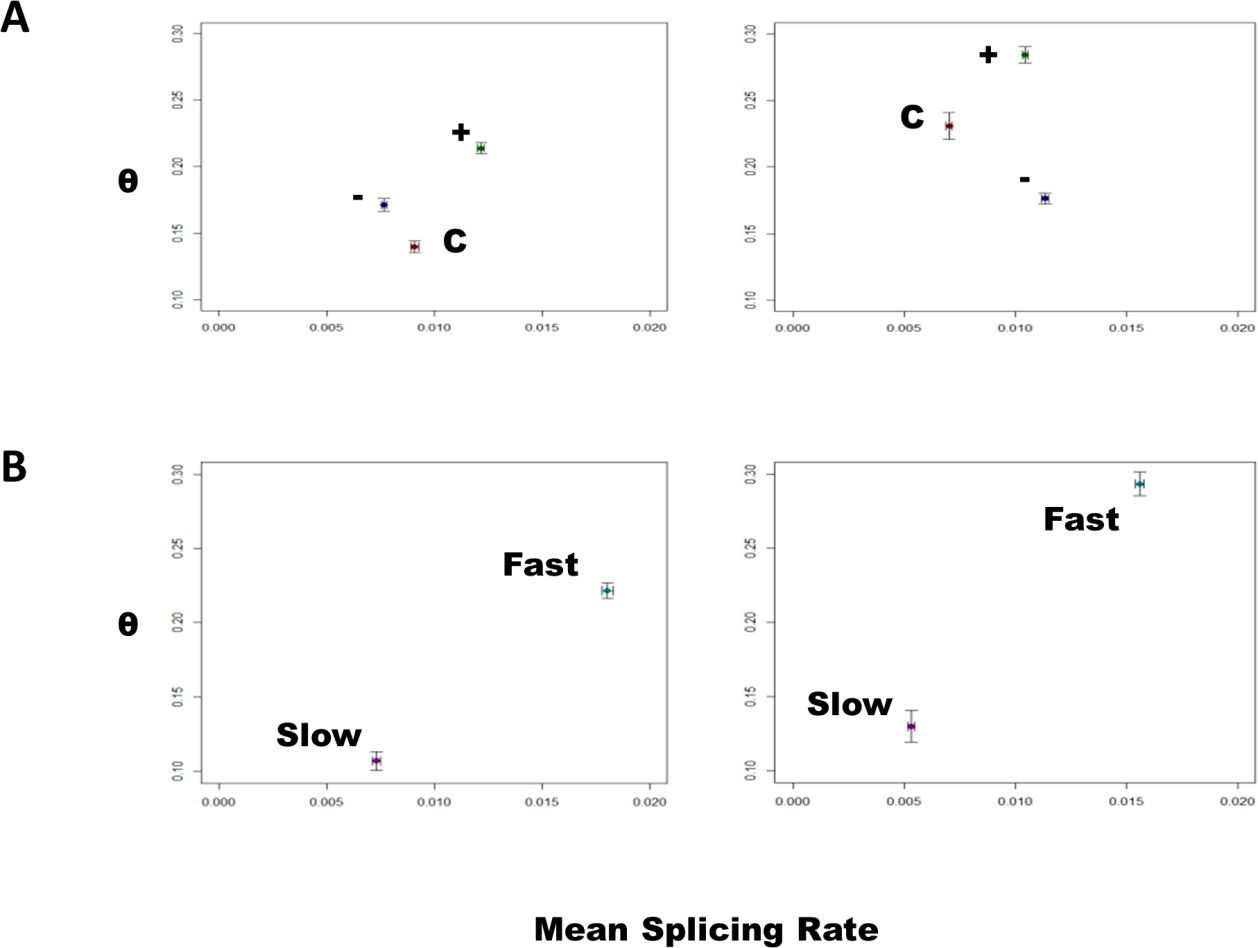
Plots of θ against the mean splicing rate with 95% confidence intervals. Exon inclusion events are shown in the left panel and exclusion inclusion events are shown in the right panel. θ is higher indicating reduced consistency of splicing in (A) *C9ORF72-ALS* (+) compared to non-*C9ORF72* ALS (-) and control (C) derived lymphoblastoid cell lines; and (B) in cell lines derived from patients with rapid (length <2 years, Fast) compared to slowly (length >4 years, Slow) progressive *C9ORF72-*ALS.

There was no significant difference in the total number of splicing events observed in lymphoblastoid cell lines derived from *C9ORF72*-ALS patients, non-*C9ORF72-*ALS patients and controls (**Fig 3**). However, the nature of those splicing events was significantly different. It is expected that functionally appropriate splicing would be similar in samples of a particular group and therefore we propose that splicing consistency is a marker of the error rate in RNA splicing. Splicing consistency was significantly reduced in the *C9ORF72*-ALS group compared to non-*C9ORF72*-ALS patients and controls (**Fig 4A, S1 Fig**). It is noteworthy that control cases do not share a common disease process and therefore might be expected to have quite different patterns of splicing. This is good evidence that splicing in *C9ORF72-ALS* is actively disrupted. In addition, splicing was less consistent in *C9ORF72-ALS* patients who lived <2 years following diagnosis compared to those that lived >4 years suggesting a link with the aggressiveness of the disease course (**Fig 4B**).

### qPCR based validation of transcriptome changes

Candidates for qPCR validation were chosen from those genes which were up-regulated in the lymphoblastoid cells derived from *C9ORF72-*ALS cases compared to controls, and also identified as candidate binding partners of the GGGGCC repeat expansion by RNA pulldown and mass spectroscopy [8]. qPCR confirmed up-regulation of *HNRNPF* (1.43 fold, t-test, p = 0.001), *RBM3* (1.18 fold, t-test, p = 0.03) and *FUS* (1.35 fold, t-test, p = 0.005) but not *HNRNPH2*.

### Estimation of expansion size and quantification of the abundance of RNA foci in lymphoblastoid cell lines

GGGGCC repeat expansion size and abundance of sense and antisense RNA foci was determined in lymphoblastoid cell lines derived from 17 patients with short (<2 years) disease duration and 7 patients with long (>4 years) disease duration. No difference in minimum (t-test, p = 0.10), modal (t-test, p = 0.41) or maximum (t-test, p = 0.57) repeat size was detectable between groups by Southern blotting (data not shown).

**Fig 5:**
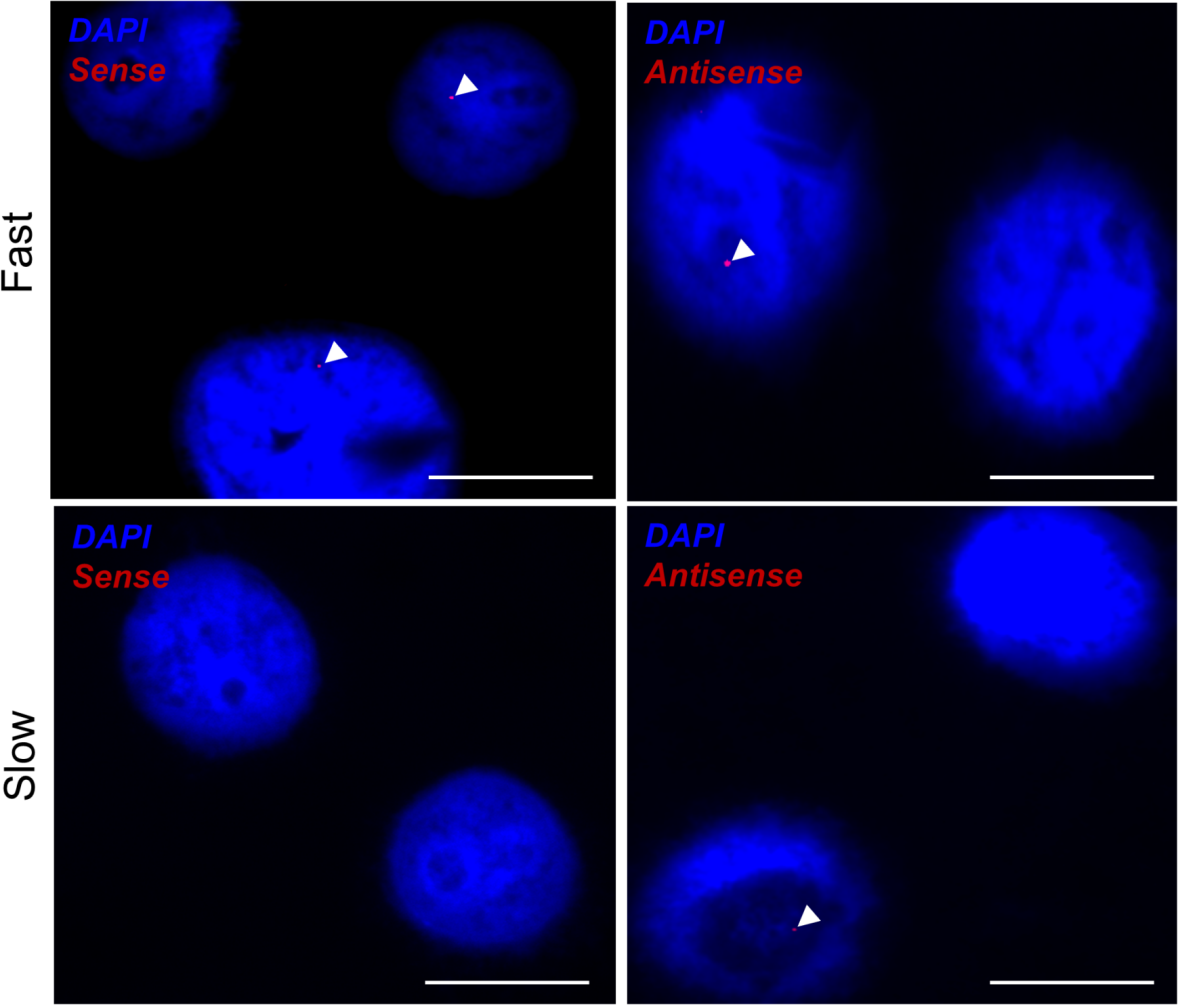
RNA foci in lymphoblastoid cell lines derived from patients with short or long survival. RNA FISH was performed for sense and antisense RNA foci in lymphoblastoid cells. Example cells are shown derived from patients with rapid (length <2 years, Fast, upper panels) compared to slowly (length >4 years, Slow, lower panels) progressive *C9ORF72*-ALS. GGGGCC-repeat sense RNA foci are visualised (arrowheads) in the left panels whereas GGCCCC-repeat antisense RNA foci are visualised (arrowheads) in the right panels. Scale bar 10 µm.

The frequency of lymphoblastoid cells containing sense RNA foci was higher in lines derived from 3 patients with short (<2 years) disease duration compared to lines derived from 3 patients with long (>4 years) disease duration, however this trend did not reach significance (average frequency of sense foci+ cells was 0.35 versus 0.12, t-test, p = 0.099). There was no such trend in the frequency of lymphoblastoid cells containing antisense RNA foci (average frequency of antisense foci+ cells 0.20 versus 0.32, t-test, p = 0.29). Example cells are shown in **Fig 5**.

## Discussion

There is an urgent need to understand the mechanisms of neuronal injury in *C9ORF72-*disease. In order to establish the biological pathways altered by the presence of the GGGGCC repeat expansion we carried out gene expression profiling of isolated motor neurons from spinal cord and lymphoblastoid cell lines derived from human ALS patients and controls. Moreover, it has been suggested that the *C9ORF72* expansion has a direct effect on the transcriptome, possibly via the formation of RNA foci [1,8]; if this is the case then transcriptome changes may represent a relatively upstream component of pathogenesis and a suitable therapeutic target.

### Transcriptome analysis in *C9ORF72*-ALS motor neurons

Six gene networks were identified as differentially expressed between *C9ORF72-ALS* and control motor neurons (**Fig 1, Table 1**). Several networks were significantly enriched for GO categories previously implicated in ALS including ‘cholesterol biosynthetic process’ which occurs primarily in the endoplasmic reticulum (ER) [14], ‘regulation of glucose metabolic process’ [15], ‘regulation of nuclear division’ [16] and ‘RNA splicing’ [17] (**Table 1**). The ‘RNA splicing’ network overlapped with a similar network in the lymphoblastoid cells and will be discussed further below.

Dysregulation of gene networks related to glucose and cholesterol metabolism, both of which were down-regulated in *C9ORF72*-ALS motor neurons, is interesting. Increasingly ER stress is implicated in ALS. As well as a site of lipid synthesis, the ER is responsible for correct protein folding [18]. Protein aggregates are a prominent feature of all forms of ALS; ER stress activates the unfolded protein response (UPR) and chronically can lead to apoptosis. Indeed activation of the UPR has been observed in sporadic ALS patients [19]. ER stress has been observed to impact negatively on cholesterol synthesis [20]. In a different cell model we have previously demonstrated a deficit in glucose metabolism associated with ALS [21].

### Transcriptome analysis in *C9ORF72*-ALS lymphoblastoid cells

Nine transcript networks were identified as differentially expressed between *C9ORF72*-ALS and control lymphoblastoid cells (**Fig 2, Table 2**). With the exception of the gene network enriched for ‘RNA splicing’, the functional enrichment of differentially expressed networks in the *C9ORF72*-ALS lymphoblastoid cells and motor neurons was distinct. This is not unexpected given the use of non-overlapping cases in the sample sets and different analysis platforms; indeed this makes the identification of a common network all the more robust. Moreover, there are differences in physiology between the cell types: motor neurons are post-mitotic whereas lymphoblastoid cells are actively dividing which may explain why ‘regulation of nuclear division’ did not appear in the lymphoblastoid cell networks.

Some common themes arise in the enrichment of the differentially expressed networks in the lymphoblastoid cells: three networks were enriched for functional categories related to nerve or muscle function (**Table 2**). This suggests that the presence of the expansion in the lymphoblastoid cells has an effect on genes important for neuromuscular function. Even if not deleterious to the lymphoblastoid cells, these same changes may be toxic if they occur in the cell types vulnerable to the neurodegenerative pathology in ALS. Two networks were enriched for functional categories related to protein catabolism (**Table 2**). Failure of protein catabolism has been implicated previously in ALS [22], indeed several genetic variants of ALS are caused by mutations in genes with roles in protein degradation e.g. VCP [23] and UBQLN2 [24]. Two networks were enriched for functional categories related to regulation of apoptosis (**Table 2**). Dysregulation of pathways related to apoptosis has also been previously implicated in ALS [25]. Both protein processing and regulation of apoptosis have been linked to ALS in the context of ER stress [18]; misfolded protein accumulation can induce ER stress, and chronic ER stress can lead to apoptosis.

A network of genes enriched for ‘RNA splicing’ as was up-regulated in both cell types under examination suggesting that it may represent an upstream effect of the expansion. Analysis of the ‘RNA splicing’ network signal in both models showed that the similarity extended beyond the functional enrichment to the actual genes dysregulated. Moreover the dysregulated gene lists were significantly enriched with independently generated candidate protein binding partners of the GGGGCC-repeat expansion from our own work [8] and that of others [7,26,27]. We have previously proposed that *C9ORF72-*disease involves dynamic sequestration of a significant number of RBPs involved in mRNA splicing, by RNA foci transcribed from the GGGGCC repeat [8]. The observed up-regulation of genes encoding these proteins in both *C9ORF72*-ALS motor neurons and lymphoblastoid cells is consistent with attempted compensation by the cell for a sequestration process.

### Exon splicing in *C9ORF72*-ALS lymphoblastoid cells

In view of these findings we attempted to examine global splicing function within the cell. Despite their being non-neuronal, we utilised lymphoblastoid cells in this analysis because of the large number of samples available and the accessibility of high quality RNA. It is reasonable to expect that a molecular phenotype observed in lymphoblastoid cells might also be present in the central nervous system (CNS). We have previously shown that detectable *C9ORF72* expansion length [28] and transcription of RNA foci [8] are comparable between lymphoblastoid cell lines and the CNS.

Splicing errors are likely to be a normal occurrence for which the cell is able to compensate [13]. However, if the load of these errors is increased then the compensatory mechanism may be overcome and the probability of this occurring might be expected to increase with time. This is consistent with the late age of onset and markedly variable phenotype found in *C9ORF72*-disease. In order to quantify splicing errors, we defined functionally appropriate splicing as likely to be consistent between members of a particular group: *C9ORF72*-ALS, non-*C9ORF72* ALS or controls. We identified a reduction in splicing consistency, or an increase in the splicing error rate, in *C9ORF72*-ALS samples compared to non-*C9ORF72* ALS samples and controls; moreover the splicing error rate was higher in samples derived from *C9ORF72*-ALS patients with shorter survival compared to samples derived from *C9ORF72*-ALS patients with a longer survival, suggesting a link with CNS toxicity. Consistent with our hypothesis there was a trend for the frequency of sense RNA foci to be higher in lymphoblastoid cell lines derived from patients with a shorter survival; this might be expected to increase the sequestration of RNA splicing proteins and thus exacerbate the production of splicing errors.

A number of studies have previously examined the transcriptome in the presence of expanded *C9ORF72* [3–5,29]; the findings of these studies have so far been inconsistent. An increase in the number of splicing errors is a potential explanation for this finding. We await further validation of our results using new technologies such as RNA sequencing and utilising newly emerging disease models including iPS derived motor neurons from patients with *C9ORF72* mutations.

## Materials and Methods

### Transcriptome analysis

#### Laser captured motor neurones

Brain and spinal cord tissue from eight *C9ORF72*-ALS patients and three neurologically normal human control subjects was obtained from the Sheffield Brain Tissue Bank (**Table 3**). *C9ORF72*-ALS samples were identified by repeat-primed PCR of the *C9ORF72* gene [1,2]. Clinically these patients resembled the full clinical spectrum of *C9ORF72*-ALS: Mean age of onset was 61 years (range 56 to 66 years) and mean disease duration was approximately 2 years (range 7 months to 43 months). Tissue donated for research was obtained with written informed consent from the next of kin, and in accordance with the UK Human Tissue Authority guidelines on tissue donation. The work was approved by the South Yorkshire Ethics Committee.

**Table 3:** Clinical information relating to motor neurons laser captured from ALS patients and controls, utilised in gene level microarray analysis.

Sample Type	Gender	Age	Duration	Diagnosis	Presentation	C9orf72
Control1	F	52	-	-	-	-
Control2	M	63	-	-	-	-
Control3	F	65	-	-	-	-
Patient1	F	62	2.00	Familial	Bulbar	+
Patient2	F	61	3.33	Sporadic	Bulbar	+
Patient3	M	66	1.17	Familial	Bulbar	+
Patient4	F	56	3.58	Familial	Limb	+
Patient5	M	62	1.67	Sporadic	Bulbar	+
Patient6	F	61	3.50	Sporadic	Limb	+
Patient7	M	70	2.17	Familial	Limb	+
Patient8	F	58	0.58	Sporadic	Limb	+

Age at symptom onset and disease duration is provided in years. Abbreviations: M = male, F = female.

Spinal cord sections from the limb enlargements were collected postmortem, processed according to standard protocols [30], and stored at −80ºC until required. Cervical spinal cord sections were prepared, between 800 and 1200 motor neurons were isolated and RNA was extracted using methods described previously [31]. RNA quantity and quality was assessed on the Nanodrop spectrophotometer and Agilent Bioanalyser, respectively, to ensure all samples were of comparable and sufficient quality to proceed. RNA (20–25ng) was linearly amplified using the Affymetrix Two Cycle cDNA synthesis protocol to produce biotin-labelled copy RNA. Copy RNA (15μg) was fragmented for 15min and hybridized to the Human Genome U133 Plus 2.0 GeneChips, according to Affymetrix protocols. Array washing and staining was performed in the GeneChip fluidics station 400 and arrays were scanned on the GeneChip 3000 scanner. GeneChip Operating Software (GCOS) was used to generate signal intensities for each transcript.

#### Data Analysis

Data were normalised using the Puma package which quantifies technical variability to improve the estimation of gene expression [32, 33]. The next step was to identify networks of genes with correlated expression which are likely to represent functional groups. To reduce the computational burden and enhance the signal strength in the data, genes were ranked by t-statistic in a disease versus control comparison; the top 10,000 genes were then taken forward. For network detection, genes were further filtered to find the 5000 most connected (as quantified by Pearson correlation coefficient) genes; by definition, networked genes are strongly connected and therefore this should not lead to loss of information [34]. Network detection was performed using the weighted gene coexpression network analysis (WGCNA) package [35]. The correlation between expression of a given network of genes and whether a sample was a *C9ORF72-*case or a control was quantified and a Student’s asymptotic p-value calculated; p-values <0.05 were taken to be significant. Differentially expressed networks were examined and an enrichment analysis performed using the Database for Annotation, Visualization and Integrated Discovery (DAVID) [36,37]. Enrichment was calculated by functional annotation clustering using the ‘high’ i.e. specific, Gene Ontology ‘biological processes’ terms.

#### Lymphoblastoid cell lines

Lymphoblastoid cell lines derived from Caucasian ALS patients (n = 56) and neurologically normal controls (n = 15), all of Northern European descent, were obtained from the UK Motor Neurone Disease Association (MNDA) DNA Bank (**Table 4**). *C9ORF72*-ALS samples were identified by repeat-primed PCR of the *C9ORF72* gene [1,2]. Clinically these patients resembled the full clinical spectrum of *C9ORF72*-ALS: Mean age of onset was 58 years (range 28 to 75 years) and mean disease duration was approximately 2 years (range 2 months to 83 months). All samples were collected with written informed consent from the donor, and the work was approved by the South Yorkshire Ethics Committee.

**Table 4:** Clinical information relating to lymphoblastoid cell lines derived from ALS patients and controls, utilised in exon level microarray analysis.

Sample Type	Gender	Age	Duration	Diagnosis	Presentation	C9orf72
Control1	F	52	-	-	-	-
Control2	M	69	-	-	-	-
Control3	F	65	-	-	-	-
Control4	F	84	-	-	-	-
Control5	M	56	-	-	-	-
Control6	F	59	-	-	-	-
Control7	M	73	-	-	-	-
Control8	F	67	-	-	-	-
Control9	M	47	-	-	-	-
Control10	M	64	-	-	-	-
Control11	F	41	-	-	-	-
Control12	M	36	-	-	-	-
Control13	M	61	-	-	-	-
Control14	M	54	-	-	-	-
Control15	F	63	-	-	-	-
Patient1	F	69	>4.00	Familial	Limb	+
Patient2	F	61	2.96	Familial	Limb	-
Patient3	F	28	1.10	Familial	Bulbar	+
Patient4	M	44	2.11	Familial	Respiratory	-
Patient5	F	46	Unknown	Familial	Bulbar	-
Patient6	M	69	1.76	Familial	Limb	+
Patient7	M	48	Unknown	Familial	Mixed	-
Patient8	M	57	5.71	Familial	Mixed	-
Patient9	F	57	1.21	Familial	Mixed	+
Patient10	M	63	>5.00	Familial	Limb	+
Patient11	F	62	0.17	Familial	Bulbar	+
Patient12	F	64	6.92	Familial	Limb	+
Patient13	M	59	<1.00	Familial	Unknown	+
Patient14	M	63	1.71	Familial	Mixed	+
Patient15	F	56	4.14	Familial	Limb	+
Patient16	M	47	1.63	Familial	Limb	+
Patient17	F	51	0.97	Familial	Bulbar	+
Patient18	F	61	Unknown	Familial	Bulbar	-
Patient19	M	73	1.88	Sporadic	Respiratory	-
Patient20	M	60	1.15	Sporadic	Bulbar	+
Patient21	M	64	2.36	Sporadic	Bulbar	-
Patient22	F	68	3.31	Sporadic	Bulbar	-
Patient23	M	68	1.56	Sporadic	Limb	+
Patient24	F	72	4.66	Sporadic	Limb	+
Patient25	M	58	1.40	Sporadic	Bulbar	-
Patient26	M	54	2.89	Sporadic	Bulbar	-
Patient27	M	53	3.28	Sporadic	Limb	-
Patient28	F	52	2.25	Sporadic	Limb	+
Patient29	M	72	2.58	Sporadic	Limb	-
Patient30	M	60	1.08	Sporadic	Bulbar	-
Patient31	F	67	1.47	Sporadic	Bulbar	+
Patient32	F	37	1.74	Sporadic	Limb	+
Patient33	M	56	2.20	Sporadic	Limb	+
Patient34	M	59	1.84	Sporadic	Limb	-
Patient35	F	70	2.13	Sporadic	Limb	-
Patient36	M	38	2.83	Sporadic	Mixed	-
Patient37	M	45	1.47	Sporadic	Limb	+
Patient38	F	48	~4.00	Sporadic	Bulbar	+
Patient39	F	72	1.87	Sporadic	Bulbar	-
Patient40	M	72	0.52	Sporadic	Limb	+
Patient41	F	75	1.05	Sporadic	Limb	-
Patient42	F	52	2.18	Sporadic	Limb	-
Patient43	F	58	1.33	Sporadic	Mixed	+
Patient44	M	47	1.57	Sporadic	Limb	+
Patient45	F	48	5.95	Sporadic	Limb	+
Patient46	M	64	0.66	Sporadic	Limb	+
Patient47	F	37	4.50	Sporadic	Bulbar	+
Patient48	M	70	1.24	Sporadic	Limb	-
Patient49	F	70	3.04	Sporadic	Limb	-
Patient50	M	61	2.57	Sporadic	Bulbar	-
Patient51	M	62	1.96	Sporadic	Limb	+
Patient52	F	58	<1.00	Sporadic	Bulbar	+
Patient53	M	61	~4.00	Sporadic	Mixed	+
Patient54	M	65	1.40	Sporadic	Limb	+

Age at symptom onset and disease duration is provided in years. Abbreviations: M = male, F = female.

Total RNA was extracted from ALS patient and control-derived lymphoblastoid cell lines using QIAGEN’s RNeasy Mini Kit following the manufacturer’s recommendations. A 75µL LCL suspension, containing approximately 5x10^6^ cells, typically yields between 1.9 and 13.6µg total RNA with a mean concentration of approximately 170ng/µl as assessed the by the NanoDrop 1000 spectrophotometer (Thermo Scientific). The quality of the isolated material was analysed using the 2100 bioanalyzer with an RNA 6000 Nano LabChip Kit (Agilent Technologies, Inc.). Linear amplification of RNA with an input of approximately 300ng of starting material was performed using the Ambion Whole Transcript (WT) Expression Assay (*Applied Biosystems*) and Affymetrix GeneChip WT Terminal Labelling Kit. This procedure generated fragments of biotin-labelled sense-stranded copy DNA (6–10µg) between 40 and 70 nucleotides in length that were hybridized onto Human Exon 1.0ST GeneChip Arrays according to Affymetrix protocols. Array washing, staining and visualisation were performed as described for motor neuron derived RNA.

#### Data analysis

Network analysis of gene expression in the lymphoblastoid cell lines was identical to that in the motor neurons. However, because of the exon level probing, after Puma normalisation, approximately twice as many transcripts were quantified. This was taken into consideration in the filtering steps and the same proportion of transcripts were analysed at each stage rather than the exact same number i.e. the top 20,000 genes ranked by t-statistic were filtered to the 10,000 most connected for the network analysis.

Exon level data were analysed using the ‘finding isoforms using robust multichip analysis’ (FIRMA) package [38] which itself is part of the Aroma Affymetrix package [39]. The FIRMA step was then applied to detect alternative splicing; a FIRMA score is calculated for each exon. The score represents the result of fitting a transcript-level model to the observed data and observing the disparity between the model and the exon-level intensity of each individual exon. Thus exons with a different level of expression to their parent transcript i.e. those which are spliced in or out, are identified. Utilising all probes specific for an entire transcript results in a significant improvement on the estimation of exon expression compared to using the relatively small number of probes specific to a given exon in isolation. FIRMA scores were log transformed prior to analysis. Highly negative or positive values of the FIRMA score are indicative of alternative exon skipping or inclusion respectively. The 1^st^ and 99^th^ percentiles of the FIRMA score for all exons in all samples were used to identify exons with the most evidence of alternative splicing, as used previously [40].

Consistency of splicing within a sample group such as patients or controls was evaluated by comparing the number of splicing events which occurred in 1, 2, 3…n samples within the group. To allow comparison between groups, comparison was made with the situation in which exons are spliced in or out at random. In each case the random situation was modelled with a Poisson distribution and the observed data were fitted to a negative binomial distribution (**S1 Fig**). θ is a quantification of the overdispersion in the negative binomial distribution with respect to an equivalent Poisson distribution, which is therefore a measure of non-random choice, i.e. consistency, in the splicing observed in each sample group. The variance of a negative binomial distribution is given by μ+μ2/θ where μ = mean. In contrast the variance of a Poisson distribution is equal to μ. Therefore a higher level of θ corresponds to a variance closer to the Poisson distribution and reduced consistency of splicing.

### qPCR based validation of transcriptome changes

Total RNA from lymphoblastoid cells was amplified using High Capacity RNA-to-cDNA kit (Applied Biosystems). Quantitative PCR (QPCR) primers for HNRNPF, FUS, HNRNPH2 and RBM3 transcripts were designed using Eurofins online primer design software (http://www.eurofinsdna.com). QPCR of 75 *C9ORF72*-ALS cases and 35 controls was performed using Brilliant II SYBR Green QPCR Master Mix (Stratagene) on the Stratagene 3000, as described previously [41]. RNA from groups of five *C9ORF72-*ALS or five control samples was pooled. These samples were obtained from the UK MNDA DNA Bank and included those samples utilised in the microarray analysis, as well as additional samples. T-tests were used to determine if the relative differences in transcript expression in lymphoblastoid cells between *C9ORF72*-ALS samples and controls were statistically significant.

### Estimation of expansion size in lymphoblastoid cell lines

GGGGCC expansion size was estimated using a Southern hybridisation based protocol as previously described [28] using DNA derived from patients with rapid (<2 years, n = 17) or slowly (>4 years, n = 7) progressive disease.

### Quantification of abundance of RNA foci in lymphoblastoid cell lines

A 5’ TYE-563-labeled LNA (16-mer fluorescent)-incorporated DNA probe was used against the sense (Exiqon, Inc.; batch number 607323) and the antisense RNA hexanucleotide repeat (Exiqon, Inc.; batch number 610331). Slides were prepared and RNA foci were visualised as described previously [8]. More than fifty lymphoblastoid cells derived from patients with rapid (<2 years, n = 3) or slowly (>4 years, n = 3) progressive disease were imaged.

## Supporting Information

S1 Fig Quantification of splicing events shared between lymphoblastoid cell lines of each sample group. Plots of the number of splicing events (y-axis) which were present in a given number of lymphoblastoid cell lines (x-axis) within a particular sample group. Sample groups from top to bottom are: normal controls, *C9ORF72-*ALS patients, non-*C9ORF72* ALS patients, *C9ORF72-*ALS patients with survival <2 years and *C9ORF72*-ALS patients with survival >4 years. In each plot the left-hand line represents a Poisson fit to the observed data i.e. the random case. The right-hand line is the observed data and the dotted line represents the negative binomial distribution fit to the observed data. In each case the negative binomial provides a relatively good fit to the observed data. θ as shown in Fig 4, is a quantification of the overdispersion in the negative binomial compared to the Poisson fit to the observed data i.e. the degree of consistency in the splicing observed in each sample group.
(TIF)

S1 Table Genes associated with the ‘RNA splicing' network signal and within the GO term ‘RNA Processing.’ Genes listed are within the GO category ‘RNA Processing’ and are significantly associated (p<0.05) with the ‘RNA splicing' network signal in either *C9ORF72*+ lymphoblastoid cell lines or motor neurons. A fold change of >1 equates to up-regulation and a fold change of <1 equates to down-regulation.
(XLSX)
