# Supplementary figures and images for: C9ORF72 GGGGCC Expanded Repeats Produce Splicing Dysregulation which Correlates with Disease Severity in Amyotrophic Lateral Sclerosis
Source: PLoS One. 2015 May 27;10(5):e0127376. doi: 10.1371/journal.pone.0127376 (PMC4446097; doi:10.1371/journal.pone.0127376)

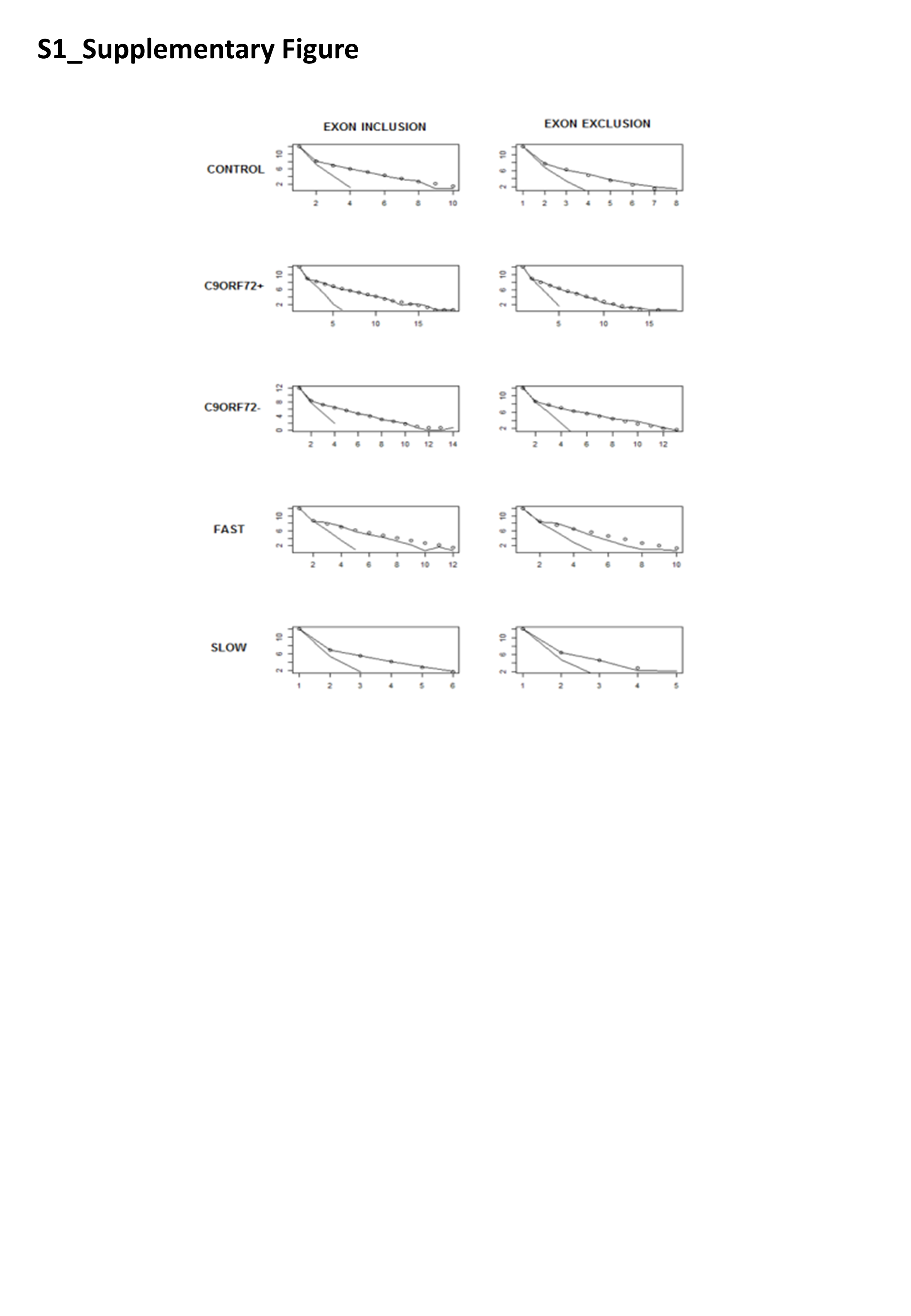

Supplement: S1 Fig — Plots of the number of splicing events (y-axis) which were present in a given number of lymphoblastoid cell lines (x-axis) within a particular sample group. Sample groups from top to bottom are: normal controls, C9ORF72-ALS patients, non-C9ORF72 ALS patients, C9ORF72-ALS patients with survival <2 years and C9ORF72-ALS patients with survival >4 years. In each plot the left-hand line represents a Poisson fit to the observed data i.e. the random case. The right-hand line is the observed data and the dotted line represents the negative binomial distribution fit to the observed data. In each case the negative binomial provides a relatively good fit to the observed data. θ as shown in Fig 4, is a quantification of the overdispersion in the negative binomial compared to the Poisson fit to the observed data i.e. the degree of consistency in the splicing observed in each sample group. (TIF) [file pone.0127376.s001.tif]
